# Central obesity may affect bone development in adolescents: association between abdominal obesity index ABSI and adolescent bone mineral density

**DOI:** 10.1186/s12902-024-01600-w

**Published:** 2024-06-06

**Authors:** Rubing Lin, Yuao Tao, Chuang Li, Feifei Li, Zijian Li, Xingyu Hong, Yantong Liu

**Affiliations:** 1 Department of Orthopedics, Shenzhen Children’s Hospital, Shenzhen, Guangdong China; 2 Department of Spine Center, Medical School, Zhongda Hospital, Southeast University, Nanjing, Jiangsu China; 3 Department of Biological Sciences, Purdue University, West Lafayette, USA; 4 Molecular Pharmacology and Experimental Therapeutics, Mayo Clinic, Rochester, USA; 5 Medical School of Chinese PLA, Beijing, China; 6 Health Service Center of Baiyun Street, Jiaojiang District, Zhejiang, 318000 China; 7 Department of Computer Information Engineering, Kunsan National University, Gunsan, 54150 South Korea

**Keywords:** Osteoporosis, A body shape index, Bone mineral density, Abdominal obesity

## Abstract

**Purpose:**

Previous studies have suggested that obesity defined by body mass index(BMI) is a protective factor for bone mineral density(BMD), but have overlooked the potential influence of different types of obesity. This study aims to evaluate the correlation between abdominal obesity index A Body Shape Index(ABSI) and adolescent bone density, and analyze the relationship between abdominal obesity and bone metabolism.

**Methods:**

A total of 1557 adolescent participants were included in NHANES from 2007 to 2018. Calculate the ABSI using a specific formula that takes into account waist circumference and BMI. A weighted multiple linear regression model is used to evaluate the linear correlation between ABSI and BMD. Forest plots are used to analyze the correlations between subgroups, and cubic splines are limited to evaluate the nonlinear correlations and saturation effects between ABSI and BMD.

**Results:**

After adjusting for confounding factors, there was a significant linear correlation (*P* < 0.01) between ABSI and femoral BMD, both as a continuous variable and an ordered categorical variable. The restrictive cubic spline curve indicates a significant nonlinear correlation and saturation effect between adolescent ABSI and BMD.

**Conclusion:**

Research has shown a significant negative correlation between ABSI and BMD at the four detection sites of the femur, and this correlation may vary slightly due to age, race, family income, and different detection sites. The research results indicate that compared to overall body weight, fat distribution and content may be more closely related to bone metabolism.

**Supplementary Information:**

The online version contains supplementary material available at 10.1186/s12902-024-01600-w.

## Introduction

Osteoporosis is a disease characterized by reduced bone mineral density(BMD) and increased fragility, and it is the most common bone disease in adults, with significant incidence and mortality rates, bringing about a considerable disease and economic burden globally [1, 2]. Childhood and adolescence are crucial periods for the accumulation of bone mass and structure, forming the foundation for strong adult bones [3]. Studies show that bone mass is likely to track from adolescence, meaning individuals will maintain their ranked positions within the distribution of a studied cohort over time [4, 5]. Therefore, BMD in children and adolescents has a significant impact on adult peak bone mass.

Obesity is a condition resulting from excessive body fat [6] and has been linked to diabetes and cardiovascular disease. It is estimated that approximately 14.4 million children and adolescents in the United States are affected by obesity [7]. Body Mass Index (BMI) is traditionally used as the primary method to assess obesity and is significantly associated with higher rates of cardiovascular disease and mortality [8]. However, many studies have shown a significant positive correlation and saturation effect between BMI and BMD [9, 10]. Some researchers believe that BMI as a marker for overall obesity cannot differentiate between general obesity and central obesity. Studies have suggested that central obesity might be negatively associated with bone development in children and adolescents [11, 12], but currently there is a lack of an effective index to assess the association between central obesity and bone density in adolescents.

The A Body Shape Index (ABSI) was designed by Krakauer NY et al [13] as a new anthropometric index independent of height, weight, and BMI. Compared with BMI, which cannot distinguish the content of fat and muscle, ABSI is a central obesity indicator that could better assess fat content and has been found to be significantly associated with various disease risks [14–17].For example, BMI, as a general obesity index, was found not to be associated with higher mortality in diabetic patients, but the abdominal obesity index ABSI showed a positive correlation [14], which showed the superiority of abdominal obesity in predicting the incidence and mortality of some diseases. In addition, ABSI has also proved a strong ability to predict cardiovascular disease in the adolescent population [18]. However, there are currently no reports on the association between ABSI and BMD in adolescents, and there is a lack of research to evaluate the relationship between abdominal obesity and bone development in adolescents, thus, a new indicator is urgently needed to establish the relationship between the two. This study aims to investigate the association between ABSI and BMD by analyzing data from the National Health and Nutrition Examination Survey (NHANES) conducted in the United States from 2007 to 2018.

## Method

### Study population

**Fig. 1:**
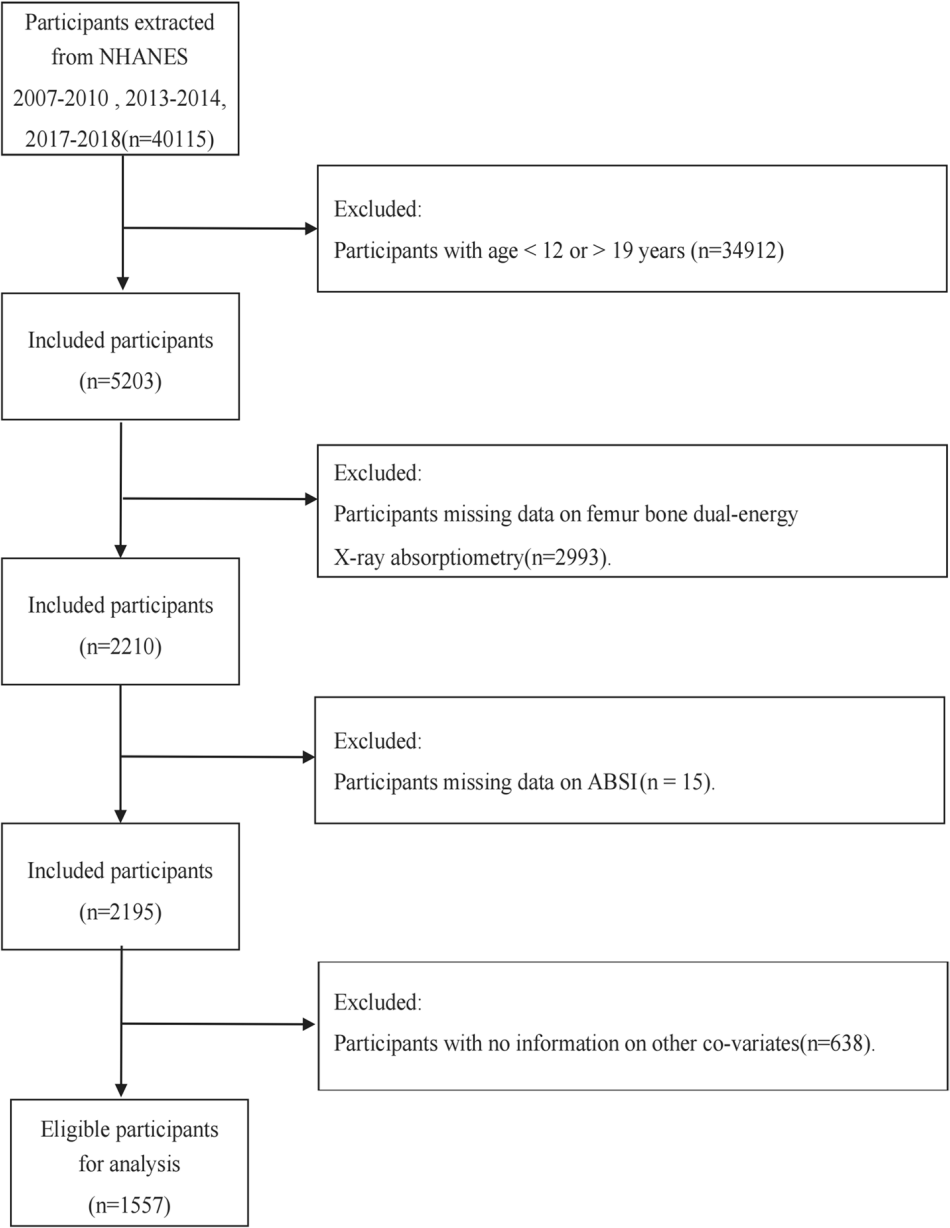
Flowchart of the sample selection from NHANES 2007–2018

The data for this study all come from the National Health and Nutrition Examination Survey conducted in the United States from 2007 to 2018 (Fig. 1). This is a nationwide study supervised and approved by the National Center for Health Statistics Ethics Review Committee, and all participants provided written informed consent. Out of the initial 40,115 participants, those who (1) were not aged between 12 and 19 years (*n* = 34,912), (2) had missing BMD data for the femur (*n* = 2993), (3) had missing ABSI data (*n* = 15), and (4) had missing data for other covariates (*n* = 638) were excluded. In the end, a total of 1,557 participants were included in this study.

### Study variables

This study included BMD measurements at four locations: the total femur, femoral neck, femoral trochanter, and the intertrochanter BMD, all obtained using a dual-energy X-ray absorptiometry (DXA) scanner. The calculation formula for the ABSI is as follows, which is calculated by dividing waist circumference(WC) by the product of BMI to the two-thirds power and the square root of height. Other covariates included:

1. Demographic variables: Age (12–19 years old), gender (male/female), race (Mexican American, Other Hispanic, Non-Hispanic White, Non-Hispanic Black, Other race), ratio of family income to poverty (PIR, < 1.3, 1.3–3.5, > 3.5). The calculation method of PIR is to divide household (or individual) income by the poverty guidelines specific to the survey year. The Department of health and human services (HHS) poverty guidelines were used as a poverty measure to calculate this ratio. PIR reflects a family’s income and higher PIR represents higher household income.
2. Diet and exercise: Energy intake (kcal/day), derived from the mean value of the two 24-hour recall dietary data. Metabolic equivalent (MET), a commonly used indicator to express relative energy metabolism levels during various activities based on energy consumption during quiet and sitting positions. The MET score is calculated by multiplying the time of each participant’s weekly activities by their relative score (Supplementary Table 1). Previous studies have shown that diet and physical activity have a great impact on adolescent bone development [19, 20], so we excluded those with missing data on daily energy intake and physical activity.
3. Laboratory data: Serum phosphorus (mg/dL), serum calcium (mg/dL), alkaline phosphatase (ALP, IU/L) - these three are commonly used indicators reflecting bone metabolism. Serum cotinine, which is a metabolite of nicotine in the blood, with a half-life of 3–5 days, can better reflect the level of nicotine exposure in the test subject, measured by an isotope-dilution high-performance liquid chromatography/atmospheric pressure chemical ionization tandem mass spectrometric method. The levels of serum phosphorus and calcium are related to bone metabolism, and ALP is a marker of bone formation [40]. Therefore, this study excluded individuals with missing relevant data.

$$\text{A}\text{B}\text{S}\text{I}=\frac{\text{W}\text{C}}{{\text{B}\text{M}\text{I}}^{2/3}{\text{h}\text{e}\text{i}\text{g}\text{h}\text{t}}^{1/2}}$$

### Statistical analysis

According to the NHANES weight selection criteria, weighted analysis was conducted for all studies except for baseline data. Student t-test is used for continuous variables, and chi square test is used for categorical variables to compare differences between ABSI quartile arrays. Use a weighted multiple linear regression model to analyze the relationship between ABSI and femur, femoral neck, femoral trochanter, and intertrochanteric BMD. Crude model, without adjusting for covariates; Model 1 was adjusted for age, gender, race, PIR; Model 2 added MET scores, serum cotinine, total energy intake as covariates to model 1; Model 3 added serum phosphorus, calcium, and ALP to model 2. After testing the significance of the linear relationship, perform a multicollinearity test, calculate the variance inflation factor (VIF) for each independent variable, ensure that there are no VIF values exceeding 10, and finally perform a normality test on the residuals to ensure that they follow a normal distribution. Subgroup analysis was conducted based on age, gender, race, PIR, and BMI, and RCS curves were used to evaluate the nonlinear relationship between ABSI and bone density at four bone detection sites in the overall population.

All analyses were performed using R software (version 4.2.1) and Excel(2308 Build 16.0.16731.20052). And statistical significance was ascertained by a two-sided *P* value < 0.05.

## Result

### Baseline characteristic

We grouped the participants into quartiles based on ABSI, as shown in Table 1. A total of 1557 adolescents participated in this study, aged between 12 and 19, with a mean age of 15.44 ± 2.23. The participants were slightly more male, accounting for 54.08% of the total. The primary race was Non-Hispanic White (33.3%). Compared to the first quartile of ABSI, those in the higher quartiles had lower femoral BMD, age, daily energy intake, and MET score, and higher ALP levels, with no significant differences in PIR, serum cotinine, serum phosphorus, or serum calcium.

**Table 1:** The baseline characteristics by ABSI: National Health and Nutrition Examination Survey 2007–2018

Characteristics	ABSI					
	Overall	Q1( < = 0.7399)	Q2(> 0.7399 & <= 0.7654)	Q3(> 0.7654 & <= 0.7963)	Q4(> 0.7963)	*p*
Number of subjects (*n*)	1557	394	384	388	391	
AGE (years, mean (SD))	15.44 (2.23)	15.86 (2.09)	15.59 (2.23)	15.16 (2.15)	15.14 (2.37)	< 0.001
GENDER						< 0.001
female	715 (45.92)	144 (36.55)	156 (40.62)	189 (48.71)	226 (57.80)	
male	842 (54.08)	250 (63.45)	228 (59.38)	199 (51.29)	165 (42.20)	
RACE (%)						< 0.001
Mexican American	413 (26.5)	59 ( 15.0)	88 ( 22.9)	116 ( 29.9)	150 ( 38.4)	
Other Hispanic	201 (12.9)	40 ( 10.2)	52 ( 13.5)	57 ( 14.7)	52 ( 13.3)	
Non-Hispanic White	519 (33.3)	107 ( 27.2)	133 ( 34.6)	136 ( 35.1)	143 ( 36.6)	
Non-Hispanic Black	340 (21.8)	171 ( 43.4)	88 ( 22.9)	55 ( 14.2)	26 ( 6.6)	
Other Race	84 ( 5.4)	17 ( 4.3)	23 ( 6.0)	24 ( 6.2)	20 ( 5.1)	
PIR (%)						0.187
<1.3	639 (41.0)	149 ( 37.8)	150 ( 39.1)	161 ( 41.5)	179 ( 45.8)	
>3.5	357 (22.9)	96 ( 24.4)	99 ( 25.8)	79 ( 20.4)	83 ( 21.2)	
1.3–3.5	561 (36.0)	149 ( 37.8)	135 ( 35.2)	148 ( 38.1)	129 ( 33.0)	
ENERGY (kcal/day, mean (SD))	2077.87 (827.67)	2194.64 (936.31)	2147.48 (802.46)	2011.85 (780.71)	1957.36 (758.33)	< 0.001
MET score (per week, mean (SD))	953.61 (997.32)	1133.56 (1143.21)	1015.57 (1002.54)	911.82 (963.56)	752.88 (815.14)	< 0.001
cotinine (ng/mL, mean (SD))	14.04 (50.65)	17.31 (56.44)	15.14 (52.86)	12.78 (47.06)	10.93 (45.37)	0.316
phosphorus (mg/dL, mean (SD))	4.36 (0.67)	4.29 (0.62)	4.36 (0.65)	4.40 (0.72)	4.40 (0.70)	0.052
calcium (mg/dL, mean (SD))	9.62 (0.31)	9.61 (0.31)	9.65 (0.32)	9.62 (0.31)	9.60 (0.30)	0.105
ALP (IU/L, mean (SD))	134.81 (92.08)	120.51 (78.56)	133.31 (95.23)	141.70 (100.12)	143.84 (91.74)	0.001
Total femur BMD (g/cm^2^, mean (SD))	1.00 (0.16)	1.08 (0.15)	1.00 (0.15)	0.97 (0.14)	0.94 (0.15)	< 0.001
Femoral neck BMD (g/cm^2^, mean (SD))	0.92 (0.15)	0.98 (0.15)	0.92 (0.15)	0.90 (0.14)	0.88 (0.14)	< 0.001
Trochanter BMD (g/cm^2^, mean (SD))	0.78 (0.14)	0.85 (0.14)	0.79 (0.13)	0.76 (0.12)	0.73 (0.12)	< 0.001
Intertrochanter BMD (g/cm^2^, mean (SD))	1.14 (0.18)	1.23 (0.18)	1.15 (0.17)	1.12 (0.17)	1.08 (0.17)	< 0.001

### Association between ABSI and bone mineral density

We evaluated the association of ABSI as both a continuous variable and as quartiles with the BMD at four measurement sites in the femur using weighted multiple linear regression models. In both crude and adjusted models for confounding factors, higher quartiles of ABSI showed significant differences in bone density compared to the first ABSI quartile group (*P* < 0.01). After adjusting for confounding factors, for every 0.1 unit increase in ABSI, the BMD at the femur, femoral neck, femoral trochanter, and intertrochanteric region decreased by 0.0553, 0.0290, 0.0611, and 0.0611 g/cm^2^, respectively. The BMD for the fourth quartile of ABSI decreased by 0.0615, 0.0319, 0.0656, and 0.0696 g/cm^2^, respectively, compared to the reference group. Additionally, there was a significant trend in the changes in BMD at the four femur sites with increasing quartiles of ABSI (Table 2).

**Table 2:** Associations between the ABSI and BMD

	ABSI		ABSI(Quartile)																
	continue, per 0.1U		Q1( < = 0.7399)		Q2(> 0.7399 & <= 0.7654)			Q3(> 0.7654 & <= 0.7963)				Q4(> 0.7963)							
Total femur BMD																			
	β(SE)	*P*-value				β(SE)	*P*-value		β(SE)		*P*-value		β(SE)		*P*-value		P for trend		
Crude Model^a^	-0.1151(0.0100)	< 0.001		Reference		-0.0710(0.0135)	< 0.001		-0.0906(0.0141)		< 0.001		-0.1270(0.0111)		< 0.001		< 0.001		
Model1	-0.0728(0.0091)	< 0.001		Reference		-0.0493(0.0113)	< 0.001		-0.0560(0.0135)		< 0.001		-0.0794(0.0097)		< 0.001		< 0.001		
Model2	-0.0688(0.0090)	< 0.001		Reference		-0.0479(0.0115)	< 0.001		-0.0538(0.0130)		< 0.001		-0.0753(0.0097)		< 0.001		< 0.001		
Model3	-0.0553(0.0100)	< 0.001		Reference		-0.0399(0.0119)	0.469		-0.0468(0.0126)		0.002		-0.0615(0.0108)		< 0.001		< 0.001		
Femoral neck BMD																			
	β(SE)	*P*-value				β(SE)	*P*-value			β(SE)	*P*-value			β(SE)	*P*-value			P for trend	
Crude Model	-0.0844(0.0093)	< 0.001		Reference		-0.0528(0.0112)	< 0.001			-0.0685(0.0137)	< 0.001			-0.0926(0.0103)	< 0.001			< 0.001	
Model1	-0.0459(0.0082)	< 0.001		Reference		-0.0323(0.0100)	0.004			-0.0366(0.0139)	0.015			-0.0493(0.0086)	< 0.001			< 0.001	
Model2	-0.0425(0.0077)	< 0.001		Reference		-0.0310(0.0100)	0.006			-0.0345(0.0134)	0.018			-0.0456(0.0085)	< 0.001			< 0.001	
Model3	-0.0290(0.0085)	0.003		Reference		-0.0232(0.0108)	0.049			-0.0278(0.0130)	0.049			-0.0319(0.0093)	0.004			0.007	
Trochanter BMD BMD																			
	β(SE)	*P*-value				β(SE)	*P*-value			β(SE)	*P*-value			β(SE)	*P*-value			P for trend	
Crude Model	-0.1072(0.0088)	< 0.001		Reference		-0.0610(0.0117)	< 0.001			-0.0839(0.0119)	< 0.001			-0.1151(0.0103)	< 0.001			< 0.001	
Model1	-0.0751(0.0087)	< 0.001		Reference		-0.0466(0.0114)	< 0.001			-0.0595(0.0117)	< 0.001			-0.0801(0.0099)	< 0.001			< 0.001	
Model2	-0.0711(0.0085)	< 0.001		Reference		-0.0453(0.0117)	0.001			-0.0571(0.0112)	< 0.001			-0.0760(0.0097)	< 0.001			< 0.001	
Model3	-0.0611(0.0090)	< 0.001		Reference		-0.0393(0.0122)	0.006			-0.0518(0.0110)	< 0.001			-0.0656(0.0104)	< 0.001			< 0.001	
Intertrochanter BMD																			
	β(SE)	*P*-value				β(SE)	*P*-value			β(SE)	*P*-value			β(SE)	*P*-value			P for trend	
Crude Model	-0.1303(0.0120)	< 0.001		Reference		-0.0837(0.0166)	< 0.001			-0.1015(0.0164)	< 0.001			-0.1462(0.0131)	< 0.001			< 0.001	
Model1	-0.0811(0.0107)	< 0.001		Reference		-0.0568(0.0127)	< 0.001			-0.0596(0.0153)	< 0.001			-0.0900(0.0112)	< 0.001			< 0.001	
Model2	-0.0768(0.0109)	< 0.001		Reference		-0.0553(0.0128)	< 0.001			-0.0573(0.0149)	0.001			-0.0857(0.0113)	< 0.001			< 0.001	
Model3	-0.0611(0.0122)	< 0.001		Reference		-0.0460(0.0132)	0.003			-0.0492(0.0145)	0.004			-0.0696(0.0128)	< 0.001			< 0.001	

Model1: Adjusted for age, gender, race, PIR

Model2: Additionally adjusted for MET scores, serum cotinine, total energy intake

Model3: Additionally adjusted for serum phosphorus, calcium, and ALP

^a^Crude model: Unadjusted model

### Subgroup analysis

**Fig. 2:**
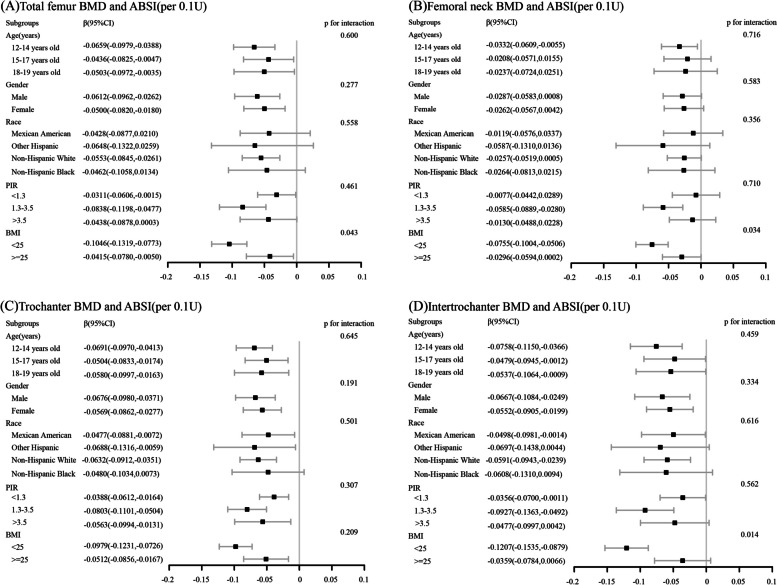
The forest plots showing the association between ABSI and femur BMD for various subgroups. **A** subgroup analysis of total femur BMD, **B** subgroup analysis of femoral neck BMD. **C** subgroup analysis of trochanter BMD, **D** subgroup analysis of intertrochanter BMD. It was adjusted for age, gender, race, PIR, MET scores, serum cotinine, total energy intake, serum phosphorus, calcium, and ALP

The forest plot (Fig. 2) shows the linear relationship between ABSI and bone density at four locations of the femur in different age, gender, race, PIR, and BMI subgroups. The results showed that in the age subgroup, the correlation between ABSI and femoral BMD was most significant in the 12–14 subgroup, with the total femur, femoral neck, femoral trochanter, and intertrochanteric region being the most significant β The values (per 0.1U) are − 0.0659 (95% CI=-0.0979, -0.0388), -0.0332 (95% CI=-0.0609, -0.0055), -0.0691 (95% CI=-0.0970, -0.0413), and − 0.0758 (95% CI=-0.01150, -0.0366), respectively. In the BMI subgroup, there is a significant correlation between ABSI and femoral BMD in the subgroup with BMI < 25, and the four sites have β The values (per 0.1U) were − 0.1046 (95% CI=-0.1319, -0.0773), -0.0755 (95% CI=-0.1004, -0.0506), -0.0979 (95% CI=-0.1231, -0.0726), and − 0.1207 (95% CI=-0.1535, -0.0879), respectively. In the subgroup analysis of gender, except for the femoral neck area, BMD in the other three areas was strongly linearly correlated with ABSI in both male and female groups.

### Analysis of non-linearity

**Fig. 3:**
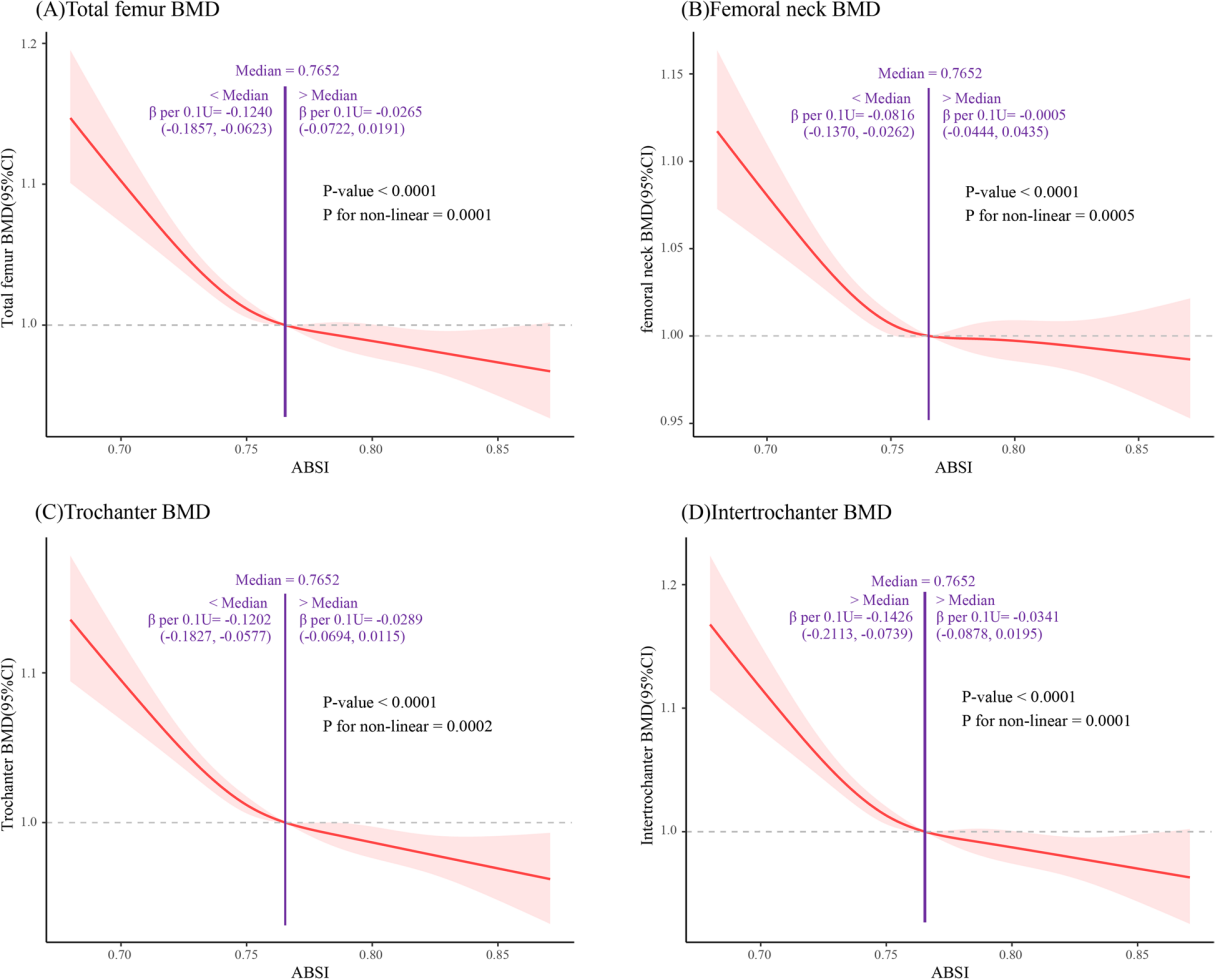
Analysis of restricted cubic spline regression. The figure shows the nonlinear relationship between ABSI and **A** total femur BMD, **B** femoral neck BMD. **C** trochanter BMD, **D** intertrochanter BMD. It was adjusted for age, gender, race, PIR, MET scores, serum cotinine, total energy intake, serum phosphorus, calcium, and ALP

In addition, we adjusted for all covariates and plotted the adjusted restricted cubic spline(RCS) curves (Fig. 3). The results indicated that the overall trends of the dependent and independent variables in the graphs were generally consistent, and individuals with higher ABSI in different groups were more likely to have lower femoral BMD. There was a non-linear association between BMD at the four femur measurement sites and ABSI. A significant non-linear association between ABSI and the risk of osteoporosis/low BMD was found in the general population and in individuals with a BMI ≥ 25 (*P* < 0.001). At the total femur, femoral neck, femoral trochanter, and intertrochanteric region, when ABSI was lower than 0.07652, for every 0.1 unit increase, the BMD at these four sites decreased by 0.1240 (95%CI=-0.1857, -0.0623), 0.0816 (95%CI=-0.1370, -0.0262), 0.1202 (95%CI=-0.1827, -0.0577), and 0.1426 (95%CI=-0.2113, -0.0739), respectively. However, when ABSI was higher than 0.07652, its association with BMD was not significant.

## Discussion

Our research findings indicate that there is a significant negative association between ABSI in adolescents and BMD at the femur, femoral neck, femoral trochanter, and intertrochanteric region. Subgroup analysis results show that the association between ABSI and BMD at these three measurement points remains significant in different subgroups of age, gender, PIR, and BMI, while the association is slightly weaker at the femoral neck site. Non-linear analysis results demonstrate a significant non-linear correlation between ABSI and femoral BMD in adolescents. When ABSI is less than 0.7652, there is a significant negative correlation between ABSI and BMD at the four femur sites; however, when ABSI exceeds 0.7652, the impact of ABSI on femoral BMD tends to saturate. Our study results illustrate a significant association between the degree of abdominal obesity in adolescents and bone density.

To our knowledge, our study is the first to explore the association between ABSI and BMD in adolescents. Previous studies have often used BMI as the standard for defining weight, leading to conclusions that overweight and obesity are protective factors for BMD [21, 22]. However, this viewpoint has been increasingly challenged. For example, Rinonapoli G et al. [23] pointed out that although an increase in BMI can lead to higher BMD, it also increases the risk of fractures. There have also been findings suggesting that obesity may have opposite effects on BMD in different parts of the body [24].A study showed that although the bone mineral content(BMC) value of obese girls was higher than that of overweight and normal weight girls, there was no significant difference in BMD among the three groups [25]. It has been suggested that BMI evaluates obesity solely based on height and weight, without considering the role of fat and muscle content [26]. Hsu et al. [27] discovered that individuals with higher fat mass are at increased risk of osteoporosis and non-spinal fractures. Guo M et al. [28] also identified a negative correlation between abdominal obesity and BMD. Hence, there is a necessity for an indicator that can effectively assess the relationship between adolescent abdominal obesity and BMD. ABSI is a new metric proposed in recent years for measuring abdominal fat, and it is independent of height, weight, and BMI. It has demonstrated strong predictive capability for cardiovascular-related disease risk. A cross-sectional study of the Chinese population discussed the association between BMI, ABSI and bone mineral density in middle-aged and elderly people [29]. The results showed that female spine BMD decreased significantly with the increase of ABSI, on the contrary, BMI showed a positive correlation with male spine BMD. Another study on the association between weight adjusted waist circumference index (WWI), an indicator of abdominal obesity in adolescents, and total bone mineral density also observed a significant negative correlation and a significant saturation effect between WWI and bone mineral density in American adolescents [30]. However, there is still no study on the association between ABSI and bone mineral density in the adolescent population. Our research findings suggest that ABSI shows a significant negative correlation with BMD in four femur regions, with a saturation effect. Additionally, based on the results of subgroup analysis, differences in the relationship between ABSI and BMD at different sites may depend on age, gender, race, PIR, and BMI subgroups. Other studies have also noted that the association between bone metabolism and influencing factors may vary by gender and race [31, 32]. However, it is likely that substantial age and race differences account for the subgroup disparities observed in assessing NHANES with BMD at different locations.In addition, sex differences may also play an important role between fat and bone parameters. A study on the association between lean and fat mass and BMD shows that for boys, lean mass is an important influencing factor of whole-body BMD, but for girls, fat mass is more important [33].

Some fundamental research aligns with the findings of this study. Marrow stromal stem cells are a common source of osteoblasts and adipocytes [34]. Obesity promotes the differentiation of stromal stem cells into adipocytes, increasing the adipocyte population in the bone marrow and decreasing osteoblast formation [35, 36]. Chronic inflammation associated with obesity is thought to play a pivotal role in maintaining the balance of bone metabolism [37]. For instance, there is a positive correlation between fat content and the expression of TNF-α, which in turn triggers NF-κB activation, thereby enhancing osteoclastogenesis by promoting the RANK and RANK ligand (RANKL) signaling pathway [38, 39]. Additionally, obesity may lead to increased leptin and decreased adiponectin release from adipose tissue, potentially increasing osteoclast activity and resulting in bone loss [40, 41]. Similar outcomes have been observed in animal studies. Chen X et al. found that rats fed a high-fat diet experienced notable loss of femoral trabecular and cortical bone, possibly due to a more substantial oxidative stress environment in obese rats causing ferroptosis in bone progenitor cells and endothelial cells [42]. ABSI represents the distribution of abdominal fat, which was used in the study to evaluate the association between central obesity and bone health, and found that central obesity may be more related to bone health than BMI, in which the content of fat and muscle played an important role. Therefore, we suggest that it is necessary to strengthen the dietary control and exercise time of adolescent groups, so as to reduce the population of central obesity among adolescents, which may be beneficial to the development of adolescent bone and improve the bone health of adolescent groups.

This study has several limitations. Firstly, as a cross-sectional study, it cannot establish a causal relationship between the ABSI and bone metabolism. Further prospective studies are needed to validate the results of this study. Secondly, data obtained through interviews in NHANES may be subject to recall bias. Finally, Some adolescent co-morbidities that have an impact on bone metabolism were not included in NHANES database, such as type 1 diabetes, celiac disease, which may have some impact on the results. In addition, this study also has several strengths, including its large scale, strong national representativeness, and stratified sampling design.

In conclusion, our research revealed a substantial negative correlation between ABSI and BMD in adolescents, as well as the saturation effect of ABSI in comparison to BMD. ABSI, independent of BMI, indicates that reducing abdominal obesity is beneficial for adolescent bone development, irrespective of the BMI-defined obesity level. Further research is required to explore potential mechanisms and interventions to alleviate the detrimental impact of abdominal fat on adolescent bone health.

## Conclusion

Our research has confirmed for the first time the negative correlation between ABSI and adolescent femoral BMD. This provides new evidence for the complex relationship between obesity and adolescent bone development, highlighting the limitations of traditional obesity measurement methods. Overall, this indicates that ABSI is an effective anthropometric measure and should be routinely measured alone or together with other indicators to improve the stratification of bone mineral density development status in adolescents. Further research is needed to elucidate the correlation between visceral fat and BMD in adolescents, as well as the potential physiological mechanisms involved.

### Supplementary Information

Supplementary Material 1.

## Data Availability

The data for this study are all from the National Health and Nutrition Examination Survey in the United States, which can be accessed through https://www.cdc.gov/nchs/nhanes/index.htm. The data for this study can also be obtained by contacting the corresponding author.

## Abbreviations

BMD: bone mineral density
NHANES: National Health and Nutrition Survey
RCS: restricted cubic splines
ABSI: A Body Shape Index
BMI: body mass index
MET score: metabolic equivalent scores
PIR: Ratio of family income to poverty

## References

[1] Gopinath V, Osteoporosis (2023). Med Clin North Am.

[2] Johnell O, Kanis JA (2006). An estimate of the worldwide prevalence and disability associated with osteoporotic fractures. Osteoporos Int.

[3] Ciancia S, Högler W, Sakkers RJB (2023). Osteoporosis in children and adolescents: how to treat and monitor?. Eur J Pediatr.

[4] Yang Y, Wu F, Winzenberg T, Jones G (2018). Tracking of areal bone mineral density from age eight to young adulthood and factors associated with deviation from tracking: a 17-year prospective cohort study. J Bone Min Res.

[5] Ma CM, Lu N, Zhang MM (2023). The relationship between obesity and bone mineral density in children and adolescents: analysis of the National Health and Nutrition Examination Survey. Arch Osteoporos.

[6] Conway B, Rene A (2004). Obesity as a disease: no lightweight matter. Obes Rev.

[7] Skinner AC, Ravanbakht SN, Skelton JA, Perrin EM, Armstrong SC (2018). Prevalence of obesity and severe obesity in US Children, 1999–2016 [published correction appears in Pediatrics. 2018;142(3):]. Pediatrics.

[8] Global BMIM, Di Collaboration E, Bhupathiraju SN (2016). Body-mass index and all-cause mortality: individual-participant-data meta-analysis of 239 prospective studies in four continents. Lancet.

[9] Wang GX, Fang ZB, Li HL, Liu DL, Chu SF, Zhao HX (2022). Effect of obesity status on adolescent bone mineral density and saturation effect: a cross-sectional study. Front Endocrinol (Lausanne).

[10] Ouyang Y, Quan Y, Guo C (2022). Saturation effect of body mass index on bone mineral density in adolescents of different ages: a population-based study. Front Endocrinol (Lausanne).

[11] Júnior IF, Cardoso JR, Christofaro DG, Codogno JS, de Moraes AC, Fernandes RA (2013). The relationship between visceral fat thickness and bone mineral density in sedentary obese children and adolescents. BMC Pediatr.

[12] Liang J, Chen Y, Zhang J (2020). Associations of weight-adjusted body fat and fat distribution with bone mineral density in Chinese children aged 6–10 years. Int J Environ Res Public Health.

[13] Krakauer NY, Krakauer JC (2012). A new body shape index predicts mortality hazard independently of body mass index. PLoS ONE.

[14] Sluik D, Boeing H, Montonen J (2011). Associations between general and abdominal adiposity and mortality in individuals with diabetes mellitus. Am J Epidemiol.

[15] Dhana K, Kavousi M, Ikram MA, Tiemeier HW, Hofman A, Franco OH (2016). Body shape index in comparison with other anthropometric measures in prediction of total and cause-specific mortality. J Epidemiol Community Health.

[16] Kuang M, Sheng G, Hu C, Lu S, Peng N, Zou Y (2022). The value of combining the simple anthropometric obesity parameters, Body Mass Index (BMI) and a Body Shape Index (ABSI), to assess the risk of non-alcoholic fatty liver disease. Lipids Health Dis.

[17] Wilczyński M, Domańska-Senderowska D, Kassassir-Ćwiklak SA, Janas Ł, Malinowski A, Wilczyński JR (2021). A Body Shape Index (ABSI) and endometrial pathology. Women Health.

[18] Mameli C, Krakauer NY, Krakauer JC (2018). The association between a body shape index and cardiovascular risk in overweight and obese children and adolescents. PLoS One.

[19] Proia P, Amato A, Drid P, Korovljev D, Vasto S, Baldassano S (2021). The impact of diet and physical activity on bone health in children and adolescents. Front Endocrinol (Lausanne).

[20] Yang LC, Lan Y, Hu J (2010). Relatively high bone mineral density in Chinese adolescent dancers despite lower energy intake and menstrual disorder. Biomed Environ Sci.

[21] Khosla S, Atkinson EJ, Riggs BL, Melton LJ (1996). Relationship between body composition and bone mass in women. J Bone Min Res.

[22] Julian V, O’Malley G, Metz L (2021). Does the severity of obesity influence bone density, geometry and strength in adolescents?. Pediatr Obes.

[23] Rinonapoli G, Pace V, Ruggiero C (2021). Obesity and bone: a complex relationship. Int J Mol Sci.

[24] Gkastaris K, Goulis DG, Potoupnis M, Anastasilakis AD, Kapetanos G (2020). Obesity, osteoporosis and bone metabolism. J Musculoskelet Neuronal Interact.

[25] El Hage R, Moussa E, Jacob C (2010). Bone mineral content and density in obese, overweight, and normal-weighted sedentary adolescent girls. J Adolesc Health.

[26] Antonopoulos AS, Oikonomou EK, Antoniades C, Tousoulis D (2016). From the BMI paradox to the obesity paradox: the obesity-mortality association in coronary heart disease. Obes Rev.

[27] Hsu YH, Venners SA, Terwedow HA (2006). Relation of body composition, fat mass, and serum lipids to osteoporotic fractures and bone mineral density in Chinese men and women. Am J Clin Nutr.

[28] Guo M, Lei Y, Liu X, Li X, Xu Y, Zheng D (2023). The relationship between weight-adjusted-waist index and total bone mineral density in adults aged 20–59. Front Endocrinol (Lausanne).

[29] Deng G, Yin L, Li K (2021). Relationships between anthropometric adiposity indexes and bone mineral density in a cross-sectional Chinese study. Spine J.

[30] Wang X, Yang S, He G, Xie L (2023). The association between weight-adjusted-waist index and total bone mineral density in adolescents: NHANES 2011–2018. Front Endocrinol (Lausanne).

[31] Xie R, Zhang Y (2023). Association between 19 dietary fatty acids intake and rheumatoid arthritis: results of a nationwide survey. Prostaglandins Leukot Essent Fat Acids.

[32] Ning HT, Du Y, Zhao LJ, Tian Q, Feng H, Deng HW (2021). Racial and gender differences in the relationship between Sarcopenia and bone mineral density among older adults. Osteoporos Int.

[33] El Hage RP, Courteix D, Benhamou CL, Jacob C, Jaffré C (2009). Relative importance of lean and fat mass on bone mineral density in a group of adolescent girls and boys. Eur J Appl Physiol.

[34] Hu L, Yin C, Zhao F, Ali A, Ma J, Qian A (2018). Mesenchymal stem cells: cell fate decision to osteoblast or adipocyte and application in osteoporosis treatment. Int J Mol Sci.

[35] Zong Q, Bundkirchen K, Neunaber C, Noack S (2023). Are the properties of bone marrow-derived mesenchymal stem cells influenced by overweight and obesity?. Int J Mol Sci.

[36] Khan AU, Qu R, Fan T, Ouyang J, Dai J (2020). A glance on the role of actin in osteogenic and adipogenic differentiation of mesenchymal stem cells. Stem Cell Res Ther.

[37] Savvidis C, Tournis S, Dede AD (2018). Obesity and bone metabolism. Hormones (Athens).

[38] Ootsuka T, Nakanishi A, Tsukamoto I (2015). Increase in osteoclastogenesis in an obese Otsuka Long-Evans Tokushima fatty rat model. Mol Med Rep.

[39] Hotamisligil GS, Arner P, Caro JF, Atkinson RL, Spiegelman BM (1995). Increased adipose tissue expression of tumor necrosis factor-alpha in human obesity and insulin resistance. J Clin Invest.

[40] Ricci R, Bevilacqua F (2012). The potential role of leptin and adiponectin in obesity: a comparative review. Vet J.

[41] Yang J, Park OJ, Kim J (2019). Adiponectin deficiency triggers bone loss by up-regulation of Osteoclastogenesis and down-regulation of Osteoblastogenesis. Front Endocrinol (Lausanne).

[42] Chen X, Liu C, Yu R (2023). Interaction between ferroptosis and TNF-α: impact in obesity-related osteoporosis. FASEB J.

