# Supplementary material for: Central obesity may affect bone development in adolescents: association between abdominal obesity index ABSI and adolescent bone mineral density
Source: BMC Endocr Disord. 2024 Jun 6;24:81. doi: 10.1186/s12902-024-01600-w (PMC11186089; doi:10.1186/s12902-024-01600-w)
Supplement: Supplementary file 1 — Supplementary Material 1. [file 12902_2024_1600_MOESM1_ESM.docx]

| **Activity(minutes per week)** | **Score(per minutes)** |
| --- | --- |
| Vigorous work-related activity | 8.0 |
| Moderate work-related activity | 4.0 |
| Walking or bicycling for transportation | 4.0 |
| Vigorous leisure-time physical activity | 8.0 |
| Moderate leisure-time physical activity | 4.0 |

Supplement table 1: MET scoring table, The total score is obtained by adding up the scores of each item
